# Time-resolved transcriptomic profiling of the developing rabbit’s lungs: impact of premature birth and implications for modelling bronchopulmonary dysplasia

**DOI:** 10.1186/s12931-023-02380-y

**Published:** 2023-03-15

**Authors:** Matteo Storti, Maria Laura Faietti, Xabier Murgia, Chiara Catozzi, Ilaria Minato, Danilo Tatoni, Simona Cantarella, Francesca Ravanetti, Luisa Ragionieri, Roberta Ciccimarra, Matteo Zoboli, Mar Vilanova, Ester Sánchez-Jiménez, Marina Gay, Marta Vilaseca, Gino Villetti, Barbara Pioselli, Fabrizio Salomone, Simone Ottonello, Barbara Montanini, Francesca Ricci

**Affiliations:** 1 Department of Experimental Pharmacology and Translational Science, R&D, Chiesi Farmaceutici S.P.A., 43122 Parma, Italy; 2 Department of Analytic and Early Formulations, Chiesi Farmaceutici S.P.A., R&D, 43122 Parma, Italy; 3 Scientific Consultancy, 48640 Bilbao, Spain; 4 Laboratory of Biochemistry and Molecular Biology, Department of Chemistry, Life Sciences and Environmental Sustainability, University of Parma, 43124 Parma, Italy; 5 Interdepartmental Research Centre Biopharmanet-Tec, University of Parma, 43124 Parma, Italy; 6 Department of Veterinary Sciences, University of Parma, 43124 Parma, Italy; 7 Institute for Research in Biomedicine (IRB Barcelona), The Barcelona Institute of Science and Technology, Baldiri Reixac, 10, 08028 Barcelona, Spain; 8 Present Address: Division of RNA Biology and Cancer, German Cancer Research Center (DKFZ), 69120 Heidelberg, Germany; 9 Present Address: Department of Medical Biotechnologies, University of Siena, 53100 Siena, Italy; 10 Head of Neonatology and Pulmonary Rare Disease, Preclinical Pharmacology, Chiesi Farmaceutici S.P.A., 43122 Parma, Italy

**Keywords:** Bronchopulmonary dysplasia, Premature birth, Transcriptomics, Proteomics, Lung development

## Abstract

**Background:**

Premature birth, perinatal inflammation, and life-saving therapies such as postnatal oxygen and mechanical ventilation are strongly associated with the development of bronchopulmonary dysplasia (BPD); these risk factors, alone or combined, cause lung inflammation and alter programmed molecular patterns of normal lung development. The current knowledge on the molecular regulation of lung development mainly derives from mechanistic studies conducted in newborn rodents exposed to postnatal hyperoxia, which have been proven useful but have some limitations.

**Methods:**

Here, we used the rabbit model of BPD as a cost-effective alternative model that mirrors human lung development and, in addition, enables investigating the impact of premature birth per se on the pathophysiology of BPD without further perinatal insults (e.g., hyperoxia, LPS-induced inflammation). First, we characterized the rabbit’s normal lung development along the distinct stages (i.e., pseudoglandular, canalicular, saccular, and alveolar phases) using histological, transcriptomic and proteomic analyses. Then, the impact of premature birth was investigated, comparing the sequential transcriptomic profiles of preterm rabbits obtained at different time intervals during their first week of postnatal life with those from age-matched term pups.

**Results:**

Histological findings showed stage-specific morphological features of the developing rabbit’s lung and validated the selected time intervals for the transcriptomic profiling. Cell cycle and embryo development, oxidative phosphorylation, and WNT signaling, among others, showed high gene expression in the pseudoglandular phase. Autophagy, epithelial morphogenesis, response to transforming growth factor β, angiogenesis, epithelium/endothelial cells development, and epithelium/endothelial cells migration pathways appeared upregulated from the 28th day of gestation (early saccular phase), which represents the starting point of the premature rabbit model. Premature birth caused a significant dysregulation of the inflammatory response. TNF-responsive, NF-κB regulated genes were significantly upregulated at premature delivery and triggered downstream inflammatory pathways such as leukocyte activation and cytokine signaling, which persisted upregulated during the first week of life. Preterm birth also dysregulated relevant pathways for normal lung development, such as blood vessel morphogenesis and epithelial-mesenchymal transition.

**Conclusion:**

These findings establish the 28-day gestation premature rabbit as a suitable model for mechanistic and pharmacological studies in the context of BPD.

**Supplementary Information:**

The online version contains supplementary material available at 10.1186/s12931-023-02380-y.

## Introduction

Extremely premature infants (born below 28 weeks of gestation) are at high risk of developing bronchopulmonary dysplasia (BPD) [1, 2]. At delivery, the lungs of extremely premature infants are still in the boundaries between the canalicular (~ 16–24 weeks of pregnancy) and saccular (~ 24–36 weeks) phases of lung development and are morphologically and biochemically immature. At this early stage of development, their lungs still lack alveolar structures and a fully mature surfactant system, which is reflected by their inability to sustain adequate gas exchange [3]. Consequently, extremely premature infants usually require intensive respiratory support to manage their respiratory distress, including assisted ventilation and supplementary oxygen [4]. Preterm birth, perinatal infections and inflammation, and life-saving therapies like post-natal oxygen and mechanical ventilation are strongly associated with the development of BPD [5]. Such triggers, alone or combined, may cause lung inflammation and alter programmed molecular patterns of normal fetal lung development, most notably normal alveolarization and pulmonary vascularization [5, 6], which clinically manifests as a chronic oxygen dependency in BPD infants.

The current knowledge on the molecular regulation of lung development mainly derives from animal studies due to the inherently limited availability of lung samples from preterm infants. Mice and rats exposed to postnatal hyperoxia or antenatal inflammation have been widely used to mimic BPD due to their limited maintenance cost, short gestation and large litter size [7, 8]. However, unlike term human neonates, who are born in the alveolar phase, term rodents are delivered in the saccular phase, displaying structurally immature but functionally mature lungs [7, 8]. Large animal models (e.g., non-human primates and lambs) provide more accurate physiological models than small laboratory animals; for instance, alveolarization starts before term birth, and their developmental timeline is comparable to humans [9]. Moreover, they can be delivered prematurely and managed with clinical-like supportive conditions [9, 10]. Nevertheless, large animal models have disadvantages such as long gestation, small litter size, high maintenance costs, and ethical limitations. Rabbit models represent a good compromise between small and large animals. They are cost-effective, have a short gestation period (31 days), have a large litter size, their lung development mirrors the human one and they can be delivered prematurely [11]. Premature rabbits delivered at 28 days of gestation (i.e., at the early saccular phase) display mild to moderate respiratory distress and impaired postnatal lung development compared to age-matched term rabbit pups [12]. The 28-day gestation preterm rabbit model exposed to postnatal hyperoxia has been used in pharmacological studies in the context of BPD [13–17].

In recent years, the combination of advanced analytical methods with bioinformatic tools has enabled high-volume analysis, integration, and interpretation of the molecular pathways involved in lung development. Microarray and proteomic technologies have been used to characterize the biomolecular aspects of lung development in murine models [18–23]. Moreover, RNA sequencing analyses have been performed on lung tissue collected at different developmental stages in mice, piglets and macaques [24–26]. Nevertheless, just a few studies conducted in mice and rhesus macaques have characterized lung developmental stages from early fetal to later postnatal phases [22, 25]. To the best of our knowledge, a time-dependent molecular characterization of the distinct stages of lung development has not yet been performed in rabbits.

The aim of the present study is to delve into the translational power of premature rabbits as a suitable model of BPD. For this purpose, we first characterized the normal lung development of the rabbit through the sequential application of histological, transcriptomic, and proteomic analyses of the different stages of lung development (i.e., pseudoglandular, canalicular, saccular and alveolar phases). Then, we investigated the impact of premature birth on the programmed molecular pathways of the lung development, comparing the transcriptomic profiles of preterm rabbits obtained during their first week of life with those from age-matched term pups. Lastly, we compared the transcriptomic results obtained in the present study with similar available records on lung development in mice [22].

## Materials and methods

### In vivo protocol and tissue collection

All experiments were approved by the intramural Animal Welfare Body and the Italian Ministry of Health (Prot. n° 744/2017-PR and n° 899/2018-PR) and comply with the European regulations for animal care. Timed pregnant rabbits (New Zealand White) were provided by Charles River Laboratories (Domaine des Oncins, France) and maintained with food and water ad libitum until the Caesarian section (C-section) or natural birth occurred (31st day of gestation).

**Fig. 1:**
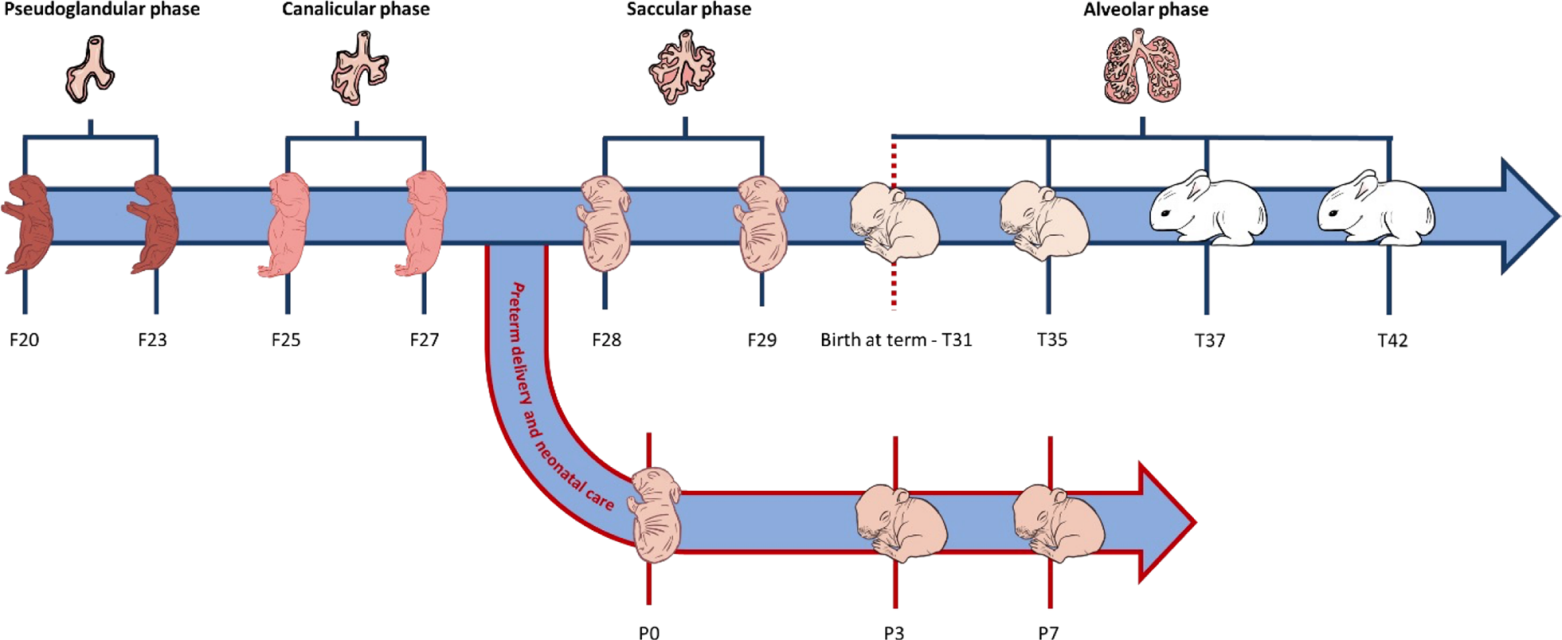
Scheme of the experimental design. Samples were collected at different lung developmental phases (i.e., pseudoglandular, canalicular, saccular and alveolar phases). Fetal (F) rabbit pups were extracted via C-section on the 20th, 23rd, 25th, 27th, 28th and 29th day of gestation (F20–F29); lung samples were harvested immediately after delivery, trying to avoid that pups took their first breath. Term (T) pups were naturally delivered on the 31st day of gestation and maintained with their mothers in room air until lung collection: immediately after birth (T31), and 4 (T35), 6 (T37), and 11 days (T42) after natural birth. Preterm (P) rabbits were delivered through C-section on the 28th day of gestation and maintained in room air until lung collection: immediately after birth (P0), and 3 (P3) and 7 (P7) days after preterm delivery

For the normal lung development study, fetal (F) pups were extracted by C-section, and the lungs were immediately harvested at the 20th, 23rd, 25th, 27th, 28th, and 29th days of gestation (n = 3–6 per time point, from F20 to F29, Fig. 1). Dams were sedated with intramuscular (i.m.) medetomidine 2 mg/kg (Domitor®, Orion Pharma, Finland). Ten minutes later, they received i.m. 25 mg/kg of ketamine (Imalgene 1000®, Merial, France) and 5 mg/kg of xylazine (Rompun®, Bayer, Germany). Subsequently, dams were euthanized with an overdose of pentothal sodium (50 mg/kg, MSD Animal Health, USA). The abdomen was immediately opened, and the uterus was exposed to extract all pups through hysterectomy. Term (T) rabbits were naturally delivered on the 31st day of gestation (T31) and maintained with their mothers at room air in individual cages up to 11 days of postnatal age. Lung samples (n = 3–6 per time point) were collected immediately after birth (T31) or 4, 6 and 11 days after birth (T35, T37, T42, respectively).

Premature (P) pups were extracted on the 28th day of gestation and kept at room air for up to 7 days. Animal care and feeding protocols have been described in detail elsewhere [12, 15]. The lung samples of premature pups were harvested 1 h (P0), 3 days (P3) and 7 days (P7) after premature delivery (n = 3 per time point, Fig. 1).

All pups were euthanized with a pentothal sodium overdose before lung harvesting. The lungs were immediately surgically dissected to avoid contact with the surrounding environment, weighted, and selectively separated into the right and left lungs. At least three lung pairs were collected at each fetal and postnatal time point, each lung obtained from pups delivered from a different dam. The right lungs were dedicated to transcriptomic analysis, while histological analysis was performed on the left ones. Exclusively for very early fetal time points (20 and 23 gestational days), transcriptomic and histological analyses were performed on whole lungs due to their reduced size. In addition, for proteomic analyses, whole lungs were harvested from littermates delivered from the same mothers of the RNA-seq pups in fetal and term groups (F25, F27, F28, F29, T31 and T35) and stored at − 80 °C.

### Lung tissue histomorphometry

Lungs were fixed with 10% formalin buffer (Sigma-Aldrich, Germany) under constant pressure (25 cmH_2_O). After 48 h, lung samples were dehydrated in graded alcohol solutions, xylene clarified, and paraffin-embedded. Serial 5 µm thick sections were obtained using a rotary microtome and stained with hematoxylin and eosin, Masson’s trichrome or orcein, according to the manufacturer’s specifications (Histo-Line Laboratories). Histological slides were acquired as whole slide images (WSI) by a digital slide scanner (Nanozoomer S-60, Hamamatsu, Japan).

Lung development was histomorphometrically analyzed by calculating the tissue density (TD), radial alveolar count (RAC), and medial thickness of pre- and intra-acinar arteries (MT%). The tissue density was determined using an application developed within the Visiopharm image analysis software (Hoersholm, Denmark). The stained tissue area of the WSI was assessed using a threshold-based mask on a lung segment, predicting the exclusion of air spaces and the inclusion of cells and nuclei. The percentage of the stained area was calculated by dividing the stained area by the total selected area (× 100). The RAC was determined by drawing a perpendicular line from the lumen of the terminal bronchiole to the nearest connective tissue septum or pleural margin, and the number of saccules or alveoli crossed by this line was counted [27, 28]. For the evaluation of MT%, ten random peripheric muscularized vessels with an external diameter (ED) of approximately 100 µm, corresponding to the pre-and intra-acinar arteries in rabbits, were selected for each section. Their external (ED) and internal diameter (ID) along the shortest axis of the vessel was measured at 40 × magnification, and MT% was calculated by applying the following formula [29]:

$${\text{MT}}\% = \left( {{\text{ED}} - {\text{ID}}} \right)/{\text{ED}} \times {1}00$$

This proportional parameter nullifies the effect of vasodilation, vasoconstriction, and tissue shrinkage.

The statistical comparisons of the histomorphometry parameters at different lung developmental phases were performed by one-way ANOVA followed by Tukey’s test (GraphPad Prism 8.4.3, San Diego, CA, USA). A *P* < 0.05 was accepted as significant.

### Transcriptomic profiling: mRNA purification, library preparation and sequencing

After collection, lungs were immediately placed in RNA Later (Sigma Aldrich, USA) and stored at − 20 °C. Samples were homogenized in QIAzol® Lysis Reagent. Total RNA was extracted with the miRNeasy Mini Kit protocol (QIAGEN, Germany), using an automated method (QIAcube: QIAGEN, Germany) adapted to include DNase I treatment. RNA concentration was measured using Qubit 4 fluorometer (Thermo Fisher Scientific, USA). RNA integrity was assessed by checking the 2:1 ratio of 18S and 28S ribosomal RNA bands and RNA Integrity Number (RIN) by agarose gel electrophoresis and Bioanalyzer RNA 6000 Nano Kit (Agilent, USA), respectively. Lung-derived RNA was suitable for RNA-sequencing (RIN > 8). Libraries for high-throughput RNA sequencing were prepared using the QuantSeq FWD kit (Lexogen, Austria) and sequenced in three different runs with the Illumina NexSeq500 platform, which generated at least 20 million reads for each sample. 96% of the reads were mapped to the rabbit genome.

### Bioinformatic analyses

Read quality analysis was performed with the *FastQC* tool. The adapters’ sequences were trimmed with *BBduk* from the suite BBtools [30]. RNA-seq reads were aligned to the rabbit genome (Oryctolagus cuniculus, OryCun2.0) with STAR [31], and gene counts were obtained with HTSeq-count [32], using the latest *Ensembl* rabbit annotation [33]. A custom script was employed to account for reads mapping to unannotated 3’ UTRs of protein-coding genes (accessible at https://github.com/danilotat/UTR_add_extend_GTF). Data are deposited at the Gene Expression Omnibus (GEO) repository under the accession number GSE220843. The batch effect due to the different sequencing runs was removed using *removeBatchEffect* function in *limma* [34]. Each sample count was normalized to the sample library size and transformed into Counts Per Million (CPM). Expressed genes were filtered using a threshold of 0.5 CPM in at least 3 samples. A Trimmed Mean of M values (TMM) normalization was applied. Differentially expressed genes (DEGs) were identified with *limma’s voom* tool (Bioconductor, R package). Genes were deemed to be differentially expressed if the absolute fold change (FC) was > 2 and the adjusted *P* ≤ 0.05.

### Gene co-expression analysis

Modules of co-expressed genes were identified using the weighted gene co-expression network analysis (WGCNA) package in R [35]. This analysis was performed on data collected from fetal and term groups. Only genes with a minimum expression of 0.5 CPM in all samples of at least 3 out of the 4 developmental phases (pseudoglandular–canalicular–saccular–alveolar) were used for module construction. Modules were identified on a signed network using the *blockwiseModules* function (WGCNA package in R) using default parameters, except for the soft thresholding power set at 10, minimum module size set at 30, and *mergeCutHeight* set at 0.25. Module eigengenes (ME) were correlated with histological parameters using Pearson correlation.

Pathway enrichment analysis was performed using Metascape software [36]. Gene Ontology Biological Processes, KEGG Pathway, Reactome, and Hallmark Gene Sets databases were used. Only terms with q-values ≤ 10^–4^ were considered significantly enriched. Heat maps were generated using the *Morpheus* tool [37]. Principal Component Analysis (PCA) was conducted using R and visualized with the package *scatterplot3D*.

### Proteomic analysis

Whole rabbit lungs from the F25, F27, F28, F29, T31 and T35 (n = 3 per time point) time points were homogenized in PBS (1 mL/100 mg) using a *GentleMax* homogenizer. Sodium dodecyl sulfate and dithiothreitol were added at final concentrations of 2% (v/v) and 0.1 M, respectively, to the lysis solution. Each sample was digested with trypsin following the filter-aided sample preparation (FASP) protocol [38]. Peptides were labelled with six-plex tandem mass tags (TMT, TMT 6-plex, Thermo Fisher Scientific) following the manufacturer’s instructions. The 18 available samples were divided into three TMT-6-plex groups, distributing one animal at each time point in each of the TMT 6-plex.

TMT sets were analyzed with the Orbitrap Fusion Lumos mass spectrometer (Thermo Fisher Scientific, Germany) coupled to a Thermo Scientific Dionex Ultimate 3000 nano RSLC, further coupled with the Advion Triversa Nanomate (Advion BioSciences, Ithaca, NY, USA) as the nanoESI source, performing nanoelectrospray through chip technology in data-dependent acquisition (DDA) mode. For the MS3 analyses for TMT quantification, multiple fragment ions from the previous MS2 scan (SPS ions) were co-selected and fragmented by higher energy collisional dissociation (HCD), using a 65% collision energy and a precursor isolation window of 2 Da. Database searches for protein identification were performed with Proteome Discoverer v2.1.0.81 software (Thermo Fisher Scientific) using Sequest HT search engine and UniProt Oryctolagus cuniculus (2020_04) and contaminants. Peptide mass tolerance was 10 ppm for the MS1 analysis, 0.6 for the MS2, and 20 for the MS3. Protein intensities were normalized using the internal reference scaling (IRS) method [39]. Briefly, only proteins detected in all samples were kept, and three normalization steps were applied: sample Loading normalization, IRS normalization, and TMM normalization.

## Results

### Distinct lung developmental stages show significantly different histomorphometry parameters

**Fig. 2:**
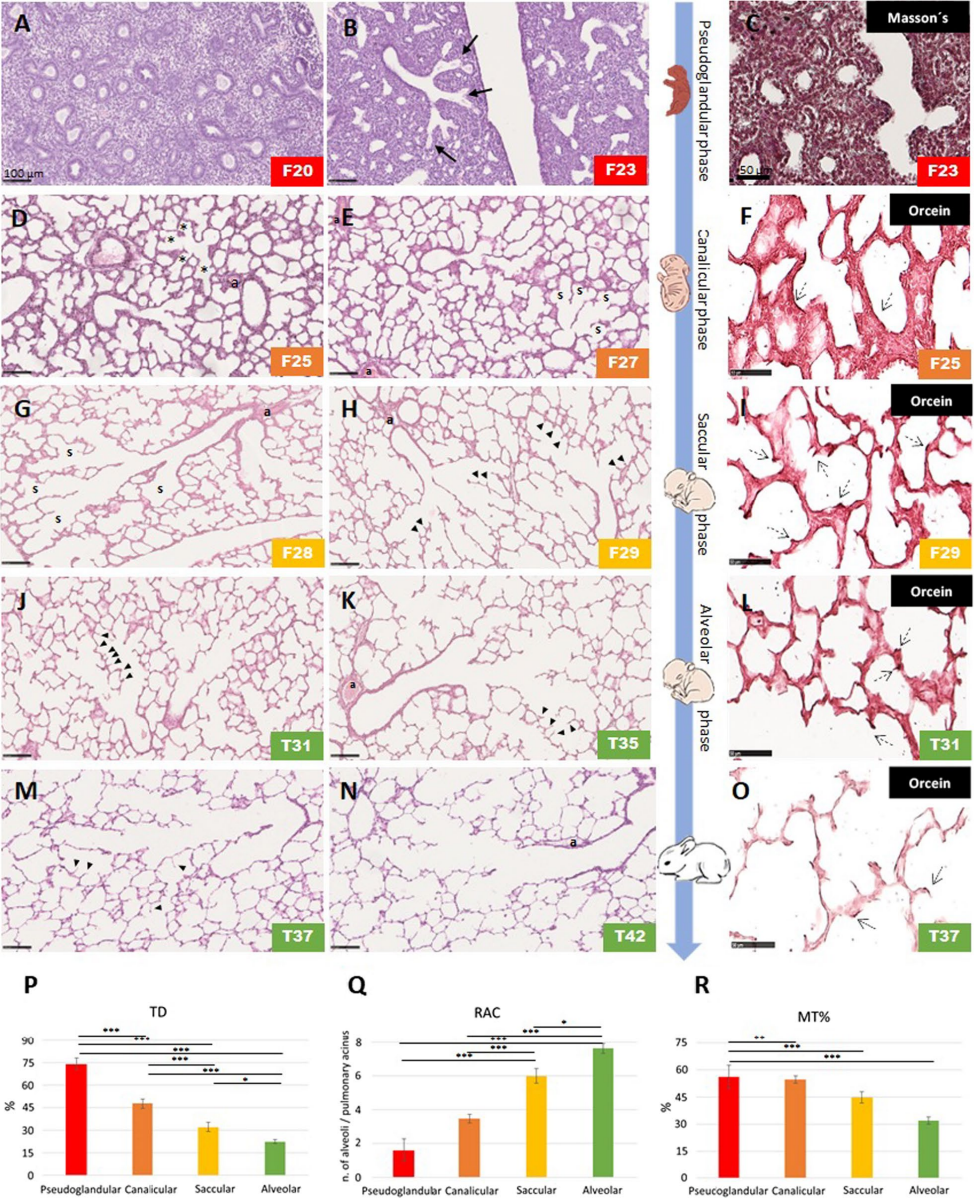
Histological characterization and histomorphometry data of the rabbit’s normal lung development. Representative microphotographs of the lung parenchyma during the pseudoglandular (**A**–**C**), canalicular (**D**–**F**), saccular (**G**–**I**) and alveolar phases (**J**–**O**) of lung development. The first two columns from the left, show sections stained with hematoxylin and eosin obtained at each lung developmental stage. In the right column, Masson’s trichrome staining (**C**) shows the scarce presence of intercellular matrix during the pseudoglandular phase, and orcein staining (**F**, **I**, **L**, **O**) shows fine elastic fibers (dotted arrows). The evolution of tissue density (TD), radial alveolar count (RAC) and the medial thickness of pre- and intra-acinar arteries (MT%) are shown in **P**, **Q** and **R** (as mean ± standard error of the mean of at least six lungs per phase of lung development. Asterisks above horizontal lines indicate a significant difference in the comparisons between different lung developmental phases (ANOVA followed by Tukey’s test; *P < 0.05; **P < 0.01; ***P < 0.001)

The normal lung development was first characterized through histological analyses and using various histomorphometry parameters. Figure 2 displays representative histological microphotographs of the pseudoglandular, canalicular, saccular and alveolar stages of the rabbit´s lung development. The pseudoglandular phase expands from F16 to F23 in the rabbit (5–17 weeks of gestation in humans and from embryonic [E] day 9.5 to E16.6 days in mice) [3, 11, 40]. At F20, the lung looked like a tubular glandular tissue (Fig. 2A), whereas airway differentiation becomes visible in F23 lungs, corresponding to the transition from the pseudoglandular to the canalicular phase. At this stage, some of the epithelial tubes at their distal end widen into airspaces covered by a more flattened epithelium than the conducting airways one (Fig. 2B, arrows). This distinction allows the recognition of the acinus/ventilatory unit for the first time.

The canalicular phase encompasses F23 to F27 (16–24 weeks of gestation in humans and E16.6 to E17.4 in mice) [3, 11, 40] and is characterized by the widening, elongation and branching of all airway generations. Simultaneously, there was a marked reduction of the pulmonary interstitial connective tissue. Each terminal bronchiole branched into 2–4 wide and straight acinar canals lined by cuboidal epithelial cells, some of which started to flatten and differentiate into type I pneumocytes (asterisks in Fig. 2D). Vascularization of the surrounding mesenchymal tissue progressively increased (Fig. 2D, E). Orcein staining revealed fine elastic fibers within the inter-canalicular septa, arranged along the perimeter of the air spaces (Fig. 2F).

The saccular phase expands from F27 to F30 (24–36 weeks of gestation in humans and E17.4 to postnatal day [PND] 5 in mice). The transition from the canalicular to the saccular phase is characterized by the branching of terminal bronchioles into several prospective alveolar ducts, ending in typical groups of enlarged air spaces (denoted by “s” in Fig. 2E, G). Terminal bronchioles appear associated with thick-walled arterioles. The mesenchymal tissue surrounding the terminal sacs condenses to form thick and highly cellular inter-saccular septa or primary septa. Few low ridges start to protrude from the primary septa to develop into secondary septa (arrowheads in Fig. 2H). Orcein staining at F29 reveals that elastic fibers, essential to elongate the crests and divide the primary saccules into smaller alveoli [41], begin to concentrate in the apical portion of the developing secondary crests (dotted arrows in Fig. 2I).

From F29 to T31, the changes in the architecture of the lung parenchyma continued but in a less pronounced way; secondary crests are visible (arrowheads in Fig. 2J) and the alveoli became more abundant, attesting to the entry into the alveolar phase. Like in humans, the alveolar phase starts in the late fetal period in rabbits (at F30) and continues postnatally (starts at 36 weeks of gestation in humans and at PND5 in mice) [3, 11, 40]. After birth, alveoli became dilated, thin-walled, and increasingly cup-shaped (Fig. 2K, M). Also, the wall of the arteries accompanying the bronchioles (pre-and intra-acinar arteries, denoted by “a” in Fig. 2N) became increasingly thin. At T31 and T36, elastic fibers appear highly localized in the apical portion of the developing secondary crests (dotted arrows in Fig. 2L, O).

The qualitative histological analysis was complemented with a semiquantitative evaluation of several histomorphometry parameters, including TD, RAC, and MT%. The progressive thinning of interalveolar septa and airspaces enlargement along the lung developmental stages was confirmed by a gradual reduction of the TD (Fig. 2P). An initial mean TD percentage of around 85% was observed in the pseudoglandular phase, which was significantly reduced in later phases (*P* < 0.001). The most abrupt and wide variation of TD was observed at the transition from the pseudoglandular to the canalicular phase (*P* < 0.001) and the transition between the canalicular and saccular phases (*P* < 0.001). Instead, the RAC increased significantly in the saccular phase compared with the pseudoglandular and canalicular phases and peaked in the alveolar phase, as expected (Fig. 2Q). The progressive thinning of the pre- and intra-acinar arterioles is denoted by the gradual decrease of MT% (Fig. 2R).

### Time-resolved transcriptomic profiling of the rabbit’s normal lung development

**Fig. 3:**
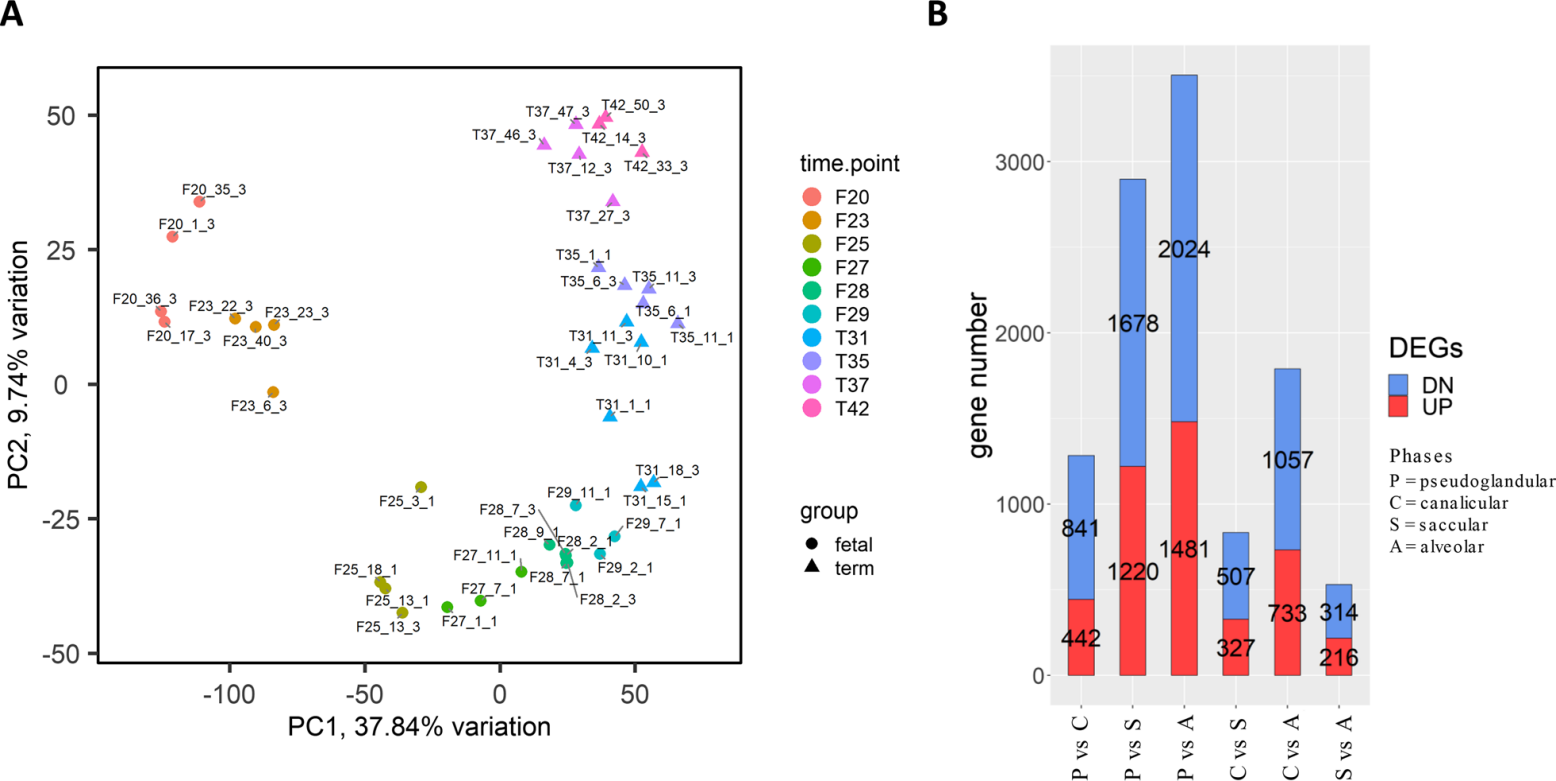
**A** Principal component analysis (PCA) highlighting specific clustering of lung samples belonging to the same time-point. The last number in sample names indicates in which RNA-sequencing run the sample was sequenced. **B** Histogram representation of differentially expressed genes (DEG) identified by comparative transcriptomic profiling of different developmental phases (P = pseudoglandular, C = canalicular, S = saccular, and A = alveolar phases). Up-regulated (UP) and down-regulated (DN) genes are shown in red and blue, respectively

A time-resolved transcriptomic analysis was performed to characterize phase-related expression patterns during lung development. In total, 12,506 protein-coding genes were identified, of which 11,482 (92%) had an identified human orthologue. Samples collected at the same lung developmental phase were grouped together after PCA analysis (Fig. 3A). Fetal samples clustered more tightly in different time points than the term ones, suggesting a progressive gene expression variation from fetal to term groups.

The limma-voom analysis identified 4160 unique genes as differentially expressed between different lung developmental phases in at least one comparison. The number of DEGs in each developmental phase comparison is reported in Fig. 3B. As expected, the number of DEGs increased with the temporal distance between the two phases considered for each comparison, and the highest number of DEGs was identified between the most distant phases (i.e., pseudoglandular vs alveolar phases). Conversely, a lower number of DEGs was detected in the comparisons between chronologically close phases (e.g., pseudoglandular vs canalicular; canalicular vs saccular; and saccular vs alveolar). The lowest number of DEGs was found for the saccular to alveolar transition.

**Fig. 4:**
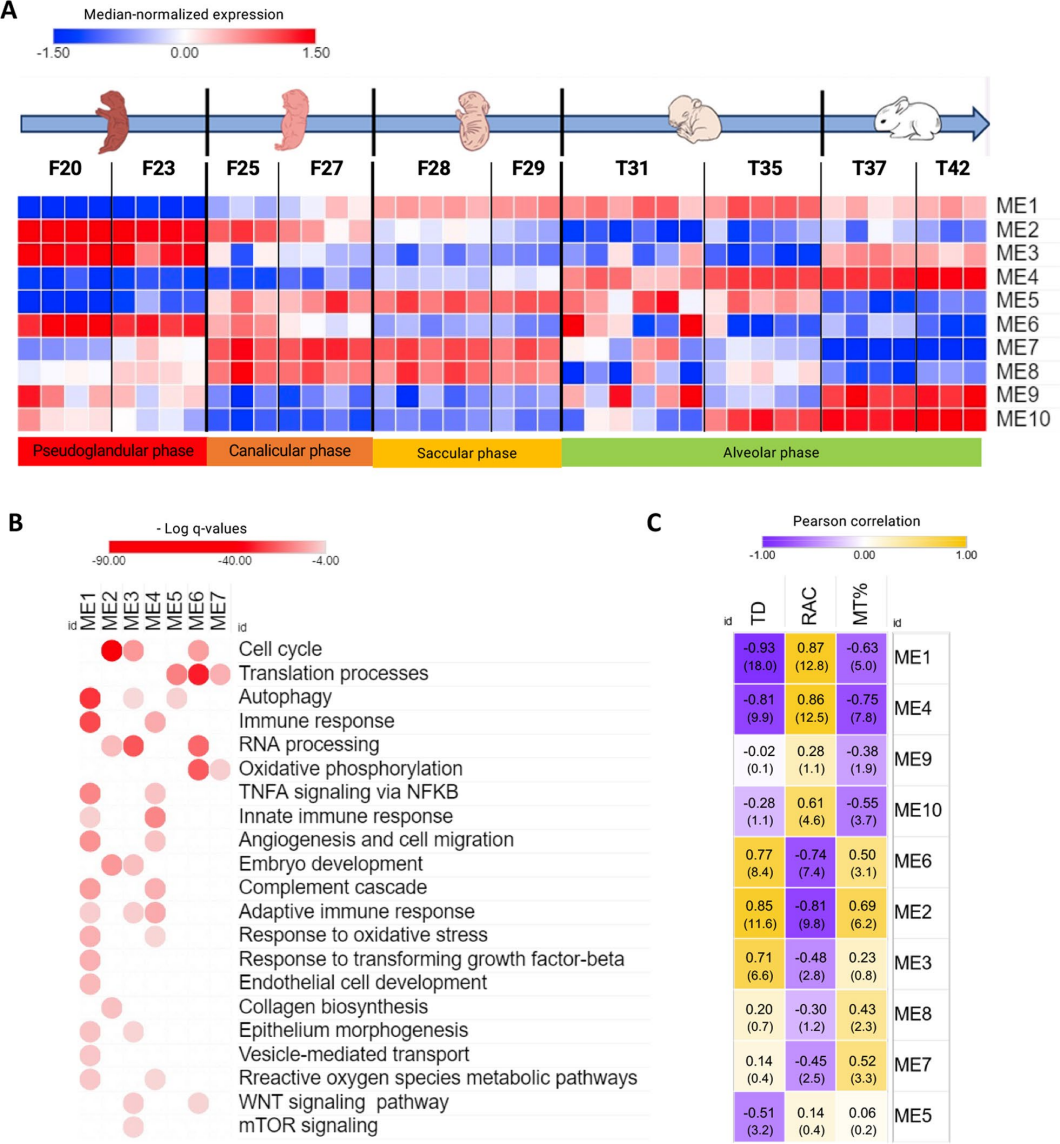
Developmental phase-dependent gene expression analysis. **A** Module eigengene (ME) heatmap representation of gene expression data derived from lung samples collected at the indicated (from fetal to post-natal) developmental time-points. Median-normalized expression levels are shown on a low-to-high scale (blue–white–red). **B** Results of pathway enrichment analysis performed on distinct modules of co-expressed genes. Representative terms, among the most significantly enriched in one or more modules, are reported (modules 8, 9 and 10 are not shown as they are enriched only in marginally statistically significant pathways; the full list of terms and modules is available in Additional file 1). Color saturation corresponds to enrichment significance (− Log q-values). C: Correlation analysis between MEs and histomorphometry parameters (negative correlations are shown in purple and positive correlations in yellow, − Log p-values are indicated in parenthesis)

Weighted gene co-expression network analysis (WGCNA) was applied in order to visualize the time-dependent modulation of gene expression. Ten modules of co-expressed genes were thus identified (Fig. 4A). For each module, gene expression appears summarized in a representative module eigengene (ME) profile (i.e., genes with similar temporal expression patterns). MEs 1–4 accounted for about 75% of all the analyzed genes. Enrichment pathway analysis was performed on genes belonging to each ME to highlight specific biological processes involved in different phases of lung development (Fig. 4B). In addition, a correlation analysis was performed between individual MEs and the outcomes of the quantitative evaluation of the histomorphometry parameters (TD, RAC, and MT%, Fig. 4C).

Module 1, which with over 2500 genes accounts for about 20% of all transcribed genes, was characterized by a gene expression trend that increased from the pseudoglandular to the alveolar phase (Fig. 4A). This module, moreover, displayed the strongest negative correlation with TD (*P* = 10^–18^). Enrichment pathway analysis revealed several pathways critical for the latest fetal phases of lung development, such as autophagy, epithelial morphogenesis, response to transforming growth factor-β (TGF-β), angiogenesis, epithelium/endothelial cells development, and epithelium/endothelial cells migration processes. In addition, pathways that might be activated as a consequence of natural birth, such as response to oxidative stress, complement cascade, and tumor necrosis factor-α (TNF-α) signaling via NF-κB, were also part of this module (Fig. 4B).

Modules 2, 3, and 6 featured an opposite trend, with high gene expression in the pseudoglandular phase and low gene expression in the later phases of lung development. Among these modules, module 2 displayed the highest positive correlation with TD (*P* = 3 × 10^–12^). Cell cycle, embryo development, epithelium morphogenesis, collagen biosynthesis, mTOR signaling, RNA processing, oxidative phosphorylation, and WNT signaling pathways were enriched in these modules related to early embryonic morphogenesis. Interestingly, almost 40% of the genes belonging to these modules code for proteins with a predicted nuclear localization, suggesting that at least a fraction of them may correspond to master regulators of lung development.

Gene expression levels in module 4 increased from birth (T31) to T42. This module also featured the highest positive correlation with RAC (*P* = 4 × 10^–13^). After birth, innate and adaptive immunity, angiogenesis, and reactive oxygen species metabolic pathways were upregulated.

Module 5 is characterized by an increase in gene expression levels from the fetal to early alveolar phase (from F25 up to T35), followed by downregulation in the late stages of the alveolar phase (T37 and T42 groups). Autophagy and vesicle-mediated transport are the most represented pathways in this module.

Modules 7 and 8 featured a marked upregulation across the canalicular and saccular phases that dampened off upon transition to the alveolar phase. Modules 9 and 10 displayed the opposite trend, with low expression levels in the canalicular and saccular phases and a subsequent upregulation with high gene expression levels in the late stages of the alveolar phase. Module 7 is enriched in translation and oxidative phosphorylation-related genes, while no significant enrichment could be detected in modules 8, 9, and 10 (see Additional file 1 for complete enrichment analysis).

### Proteomic validation of the transcriptomic profiling of the normal rabbit’s lung development

A quantitative proteomic analysis was performed to characterize the proteome profile and validate the transcriptomic analysis of the canalicular, saccular and alveolar stages. Collectively, 28,427 peptide groups were generated, which mapped 4367 proteins, of which 2164 proteins were observed in all analyzed samples.

**Fig. 5:**
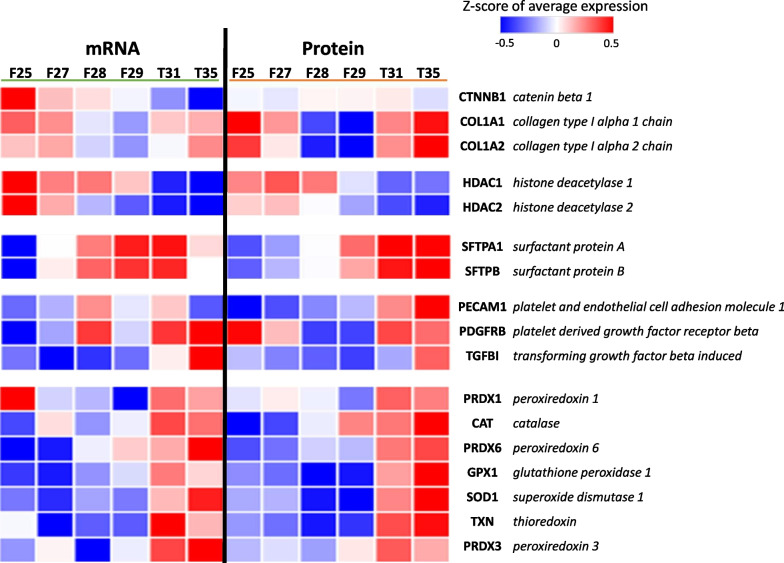
Comparison of the expression at mRNA and protein levels for selected genes of interest. Z-score-normalized expression levels are indicated on a low-to-high scale (blue–white–red)

Integration of proteomic and transcriptomic profiles revealed 1927 genes common to both analyses. A subset of genes was selected in order to validate the observed gene expression with the encoded protein levels during different lung developmental stages (a manuscript with the full report of the proteomic analysis is under preparation). Histone deacetylases 1 and 2 (HDAC1 and HDAC2) were more expressed both at the transcriptional and proteomic levels from F25 to F27 (Fig. 5). The expression of surfactant proteins A and B (SFTPA1 and SFTPB) increased from F28 with higher protein upregulation from F29 along the alveolar phase. Catenin beta 1 (CTNNB1) and collagen type I alpha 1 and alpha 2 chain proteins (COL1A1 and COL1A2) are mostly expressed in the alveolar phase. Peroxiredoxin 1, 3 and 6 (PRDX1, PRDX3. PRDX6), catalase (CAT), glutathione peroxidase (GPX1), superoxide dismutase 1 (SOD1), thioredoxin (TXN), platelet and endothelial cell adhesion molecule 1 (PECAM), platelet derived growth factor beta (PDGFRB) and transforming growth factor beta induced (TGFBI) showed a similar expression pattern between protein and mRNA, being mostly expressed in the alveolar phase.

### Premature birth induces distinct gene expression patterns in the developing lung compared with the normal lung development

**Fig. 6:**
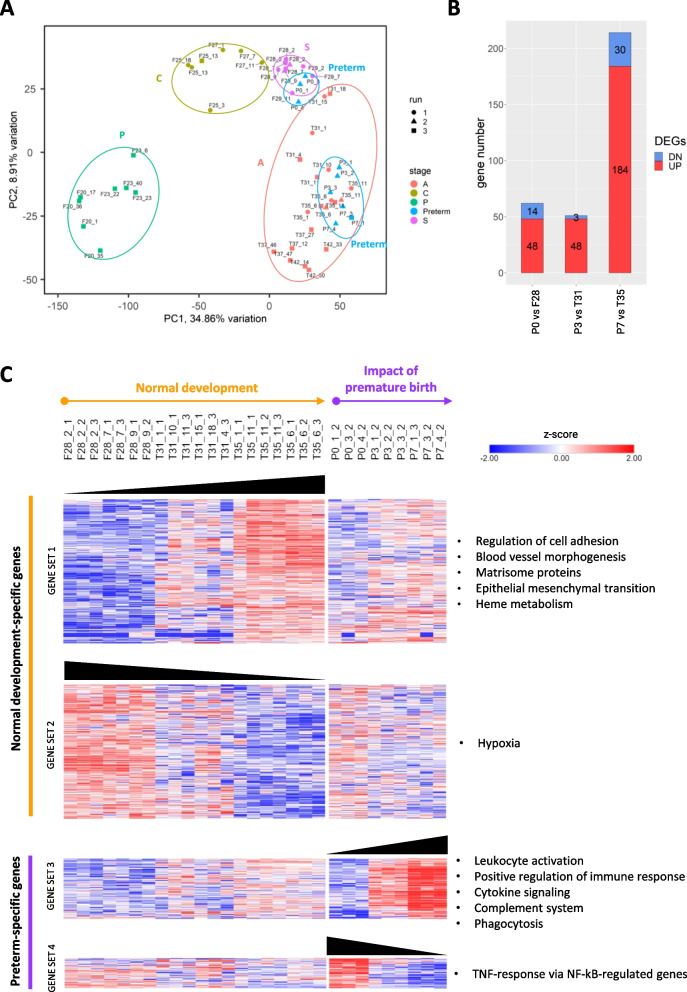
The impact of premature birth on the molecular pathways of lung development. **A** Principal component analysis (PCA) shows preterm pups clustering closely to, yet well separated from, term pups of the same gestational age. Samples were sequenced in three different RNA-sequencing runs and batch-effect correction was applied. **B** Number of differentially expressed genes between preterm rabbit pups and age-matched term pups at different time points. Upregulated and downregulated genes are shown in red and blue, respectively. **C** Heatmap representing expression levels during normal development or after premature birth. Gene set 1 and gene set 2 refer to genes and pathways respectively upregulated and downregulated in term pups but not modulated in preterm pups; gene set 3 and gene set 4 refer to genes and pathways respectively upregulated and downregulated in preterm pups but not modulated in term pups. The last number in sample names indicates in which RNA-sequencing run the sample was sequenced. Z-score-normalized expression level is indicated on a low-to-high scale (blue–white–red). Main pathways enriched in each gene set are indicated on the right

The impact of premature birth on gene expression was evaluated by comparing the transcriptomic profiles of preterm (P0 at 1 h, P3 and P7) and age-matched pups for their physiological lung development (F28, T31 and T35) (Fig. 6A). Upregulated genes outnumbered downregulated genes in all comparisons, ranging from 71.7% to 86.0% of the total DEGs (62 genes in P0 vs F28, 51 in P3 vs T31, and 214 in P7 vs T35 comparisons, respectively) (Fig. 6B). Nevertheless, when considering age-matched pups, genes up-regulated in the P7 vs T35 comparison could either be due to a decrease of gene expression during normal development or to an increase in preterm pups. Therefore, DEG lists were created for P0 *vs* F28, P3 *vs* T31 and P7 *vs* T35 comparisons, which were combined with lists of dysregulated genes during normal lung development (i.e., genes dysregulated in the F28 vs T35 comparison) but not dysregulated after preterm birth (P0 vs P7 comparison) and vice versa (Fig. 6C).

Pathway enrichment analysis revealed a prevalence of upregulated immune system-related genes and pathways in preterm animals compared to age-matched term pups. The significance of these pathways increased as a function of post-natal time (i.e., from P3 *vs* T31 to P7 *vs* T35 comparisons) and moreover were specifically upregulated only in preterm pups (i.e., P7 *vs* P0 comparison, gene set 3). Notably, regarding the P0 *vs* F28 comparison, up-regulated genes were significantly enriched in TNF-responsive, NF-κB-regulated genes. Other genes characterized by downregulation during normal development, but not downregulated in preterm pups were enriched in hypoxia pathway-related genes. Genes involved in cell adhesion, heme metabolism, blood vessel morphogenesis, extracellular matrix protein expression (matrisome proteins) and epithelial-mesenchymal transition processes were exclusively upregulated in term animals but not in premature pups (gene set 1, T35 *vs* F28 comparison).

### Comparison of the rabbit and mice lung transcriptomes between late fetal stage and term birth

**Fig. 7:**
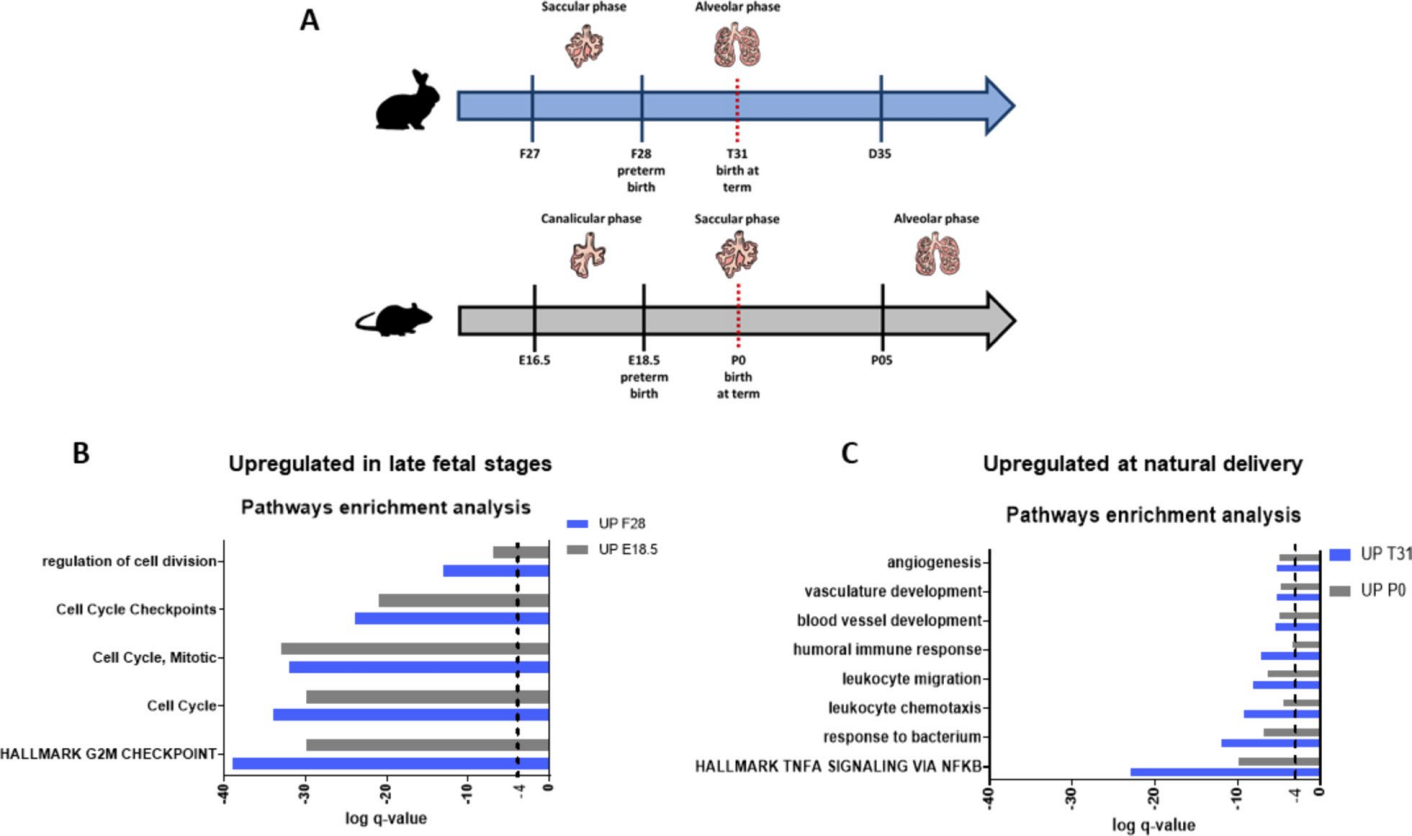
Developmental gene expression comparison between rabbits and mice. **A** Comparison between rabbit and mouse physiological lung development. Rabbits are born at term (T31) in the alveolar phase, whereas mice are born at term (PND0) in the saccular phase. Mice enter the alveolar phase only 4–5 days after birth. **B** Up-regulated pathways in rabbits (blue line) and mice (grey line) at preterm birth (F28 and E18.5 time points, respectively). **C** Up-regulated pathways in rabbits (blue line) and mice (grey line) at term birth (T31 and PND0 time points, respectively). The identification of processes enrichment for each comparison was performed using the Metascape software. Only processes with q-values ≤ 10^–4^ were considered significantly enriched

An independent dataset available from Beauchemin et al*.* (GEO: GSE74243) [22] was used to compare mouse and rabbit lung developmental transcriptomic profiles. Specifically, DEG lists comparing E18.5 *vs* PND0 in mice (intra-saccular phase comparison) and F28 *vs* T31 in rabbits (saccular vs alveolar) were created and subsequently used for pathways enrichment (Fig. 7A). Characteristic pathways of organ development such as cell cycle, regulation of cell division, cell cycle checkpoint, and mitotic processes were upregulated in both mice (E18.5) and rabbits (F28) at late fetal lung development, with comparable Log q-values for each pathway in both species (Fig. 7B). At term (PND0 in mice and T31 in rabbits) both species were enriched in angiogenesis, vasculature development, blood vessel development, humoral immune system, leukocyte migration, and TNFα signalling via NF-κB even if they born at term in different lung developmental phases (Fig. 7C).

## Discussion

Due to the intrinsic limitations in obtaining representative samples from BPD infants, animal models are essential to decipher the intricate molecular pathways involved in BPD. Rats, mice, rabbits, lambs, and non-human primates have been used to recapitulate the pathophysiology of BPD, incorporating into the animal models well-known clinical “triggers” of BPD such as postnatal hyperoxia, perinatal infections and inflammation, and invasive mechanical ventilation [8–11]. Interestingly, however, less attention has been placed on characterizing the impact of premature birth (i.e., without further perinatal insults) on the molecular regulation of lung development, even though it is a common risk factor in all BPD infants. To fill this knowledge gap, we took advantage of the rabbit model, which represents a cost-effective alternative to non-human primates and enables the delivery of premature rabbit pups with respiratory distress.

In the first place, we applied histomorphometry and histological analyses to investigate the progression of the rabbit’s normal lung development. Histomorphometry data indicated a high level of significance for TD and MT% in the pseudoglandular-to-canalicular and canalicular-to-saccular transitions, whereas the changes in TD were less significant in the saccular-to-alveolar transition, without significant differences for MT%. Similarly, the striking difference between the saccular phase and the previous pseudoglandular and canalicular phases in terms of RAC was less pronounced (yet still significant, P < 0.05) in the transition from the saccular to the alveolar phase. This is in line with the histological observation of less evident changes in the lung parenchyma between the saccular and alveolar phases. Overall, the morphological characterization of the normal rabbit’s lung development allowed clear differentiation of the distinct developmental stages, thereby validating the selected time-points as stage-specific for the transcriptomic and proteomic analyses.

Next, we performed a time-resolved transcriptomic and proteomic characterization of the normal rabbit’s lung development. Here, the proteomic data consist of a panel of proteins of interest with the purpose of validating the findings from the transcriptomic profiling. Notably, proteins and transcripts obtained from independent experiments showed a fairly common expression pattern. The transcriptomic analysis demonstrated marked differences in the type and number of dysregulated genes along different developmental stages, with the lowest number of dysregulated genes observed for the comparison between the saccular and alveolar phases. The gene expression patterns and pathways involved during physiological lung development were determined by applying WGCNA analysis, which has been recently used to characterize and identify key pathways involved in several pathologies, including BPD [42–44]. Pathway enrichment analysis on co-expressed gene modules identified different expression patterns during normal lung development. For instance, cell cycle and embryo development-related pathways (genes in ME2 and ME3) appear upregulated in the first developmental phases (up until F27), a sign of organ expansion and early embryonic morphogenesis processes.

The genes in the ME1 are characterized by a low expression in the pseudoglandular and canalicular phases and their upregulation in the saccular phase that persists during the alveolar phase. Pathway enrichment analysis of these genes revealed molecular processes that have been described to be essential to specialize the lungs for the extrauterine transition, including autophagy, FOXO-mediated transcription, epithelium/endothelium development pathways, and TGF-β response, among others [45–49]. Interestingly, these pathways increase their expression at F28, corresponding with the starting point for the 28-day gestation premature rabbit model of BPD [13, 14, 16]. Furthermore, the respiratory distress observed in the 28-day gestation rabbit model [12] correlates well with our analysis, showing that the expression of SFTPA1 and SFTPB mRNAs and their subsequent translation started at F28, which remained upregulated during the alveolar phase. Surfactant production and innate immune responses are critical and connected processes necessary for breathing and survival after birth [50]. Our results also show an upregulation of the immune system, response to oxidative stress, complement, and TNF-α signaling pathways in the transition between the saccular and alveolar stages during normal lung development.

Initiation of breathing activates critical metabolic and cardiorespiratory changes in the newborn to adapt the lungs to the new environment [51]. In this regard, the genes of ME4, which are characterized by an increased expression pattern after birth (T31), show an upregulation of the reactive oxygen species (ROS) metabolic processes and angiogenesis and cell migration (vascular endothelial growth factor [VEGF] signaling). Accordingly, the proteomic analysis revealed that antioxidant enzymes (PRDX1, CAT, PRDX6, GPX1, SOD1, TXN, and PRDX3) are highly expressed and translated at term (T31). Moreover, the proteomic analysis included other proteins that are mainly expressed during late lung development. For instance, PECAM plays a role in angiogenesis and is important for the process of alveolar septation; the loss of PECAM results in increased endothelial sensitivity to apoptotic stress, an altered response in the recruitment of neutrophils, and decreased angiogenesis [52]. PDGFRB is a tyrosine-protein kinase involved in regulating embryonic development and cell proliferation; its receptor also plays an essential role in blood vessel development by promoting proliferation, migration, and recruitment of pericytes and smooth muscle cells to endothelial [53]. TGFBI is involved in early alveolarization and in pulmonary angiogenesis; this downstream target of TGF-β signaling is a crucial component of the distal lung extracellular matrix, which is necessary for normal alveolar secondary septation [54, 55].

Premature birth is associated with significant respiratory morbidity in human preterm neonates. Respiratory distress syndrome (RDS) and BPD are the most prevalent pulmonary conditions affecting premature neonates, and their incidence increases with decreasing gestational age [5, 56]. RDS is primarily caused by a severe surfactant deficiency and is the consequence of incomplete lung maturation at delivery. In the case of BPD, premature birth represents the starting point of the disease, which develops postnatally in response to perinatal inflammatory events that activate not yet fully understood molecular mechanisms of lung injury and repair that disrupt normal lung development [5]. Premature rabbits closely mimic the lung immaturity observed in human neonates. Rabbit pups delivered after 27 days of gestation suffer from severe RDS and show a very limited life span (i.e., a few hours) despite receiving mechanical ventilation and surfactant treatment [57–59]. After 28 days of gestation, premature rabbits can breathe spontaneously and display a moderate RDS at birth. Salaets et al*.* have recently compared the pulmonary outcomes of premature rabbits delivered at 28 days of gestation and maintained in room air for seven days with age-matched term controls [12]. Their results indicated that prematurity per se may cause delay in lung development, even in the absence of hyperoxia or mechanical ventilation. Motivated by this study, we performed a transcriptomic analysis using a similar experimental design to investigate the impact of premature birth on pulmonary molecular regulation.

Our analysis reveals that premature birth causes a significant dysregulation of the inflammatory response. Remarkably, TNF-responsive, NF-κB regulated genes were significantly upregulated just one hour following premature delivery. NF-κB downstream target genes include innate and adaptive immune response factors, such as cytokines, chemokines, and cell adhesion molecules [60]. Hence, the upregulation of TNF-responsive, NF-κB regulated genes immediately after premature birth seems to trigger the upregulation of downstream inflammatory pathways such as leukocyte activation and cytokine signalling that persist during the first week of life. The analysis also revealed genes that appear dysregulated during normal lung development but are not dysregulated in premature animals, including genes involved in blood vessel morphogenesis, epithelial-mesenchymal transition and matrisome proteins (i.e., genes encoding for the extracellular matrix and associated proteins). Conversely, hypoxia genes were significantly downregulated only in term pups. The differential regulation of hypoxia genes in the early postnatal life may be explained by hypoxic episodes in premature rabbits due to their respiratory distress. Hypoxic episodes are relatively common in premature neonates due to their immature respiratory control [61].

Lastly, we compared our transcriptomic data on the rabbit´s lung development with the transcriptomic study conducted by Beauchemin et al. in mice [22]. Newborn rodents exposed to postnatal hyperoxia or perinatal inflammation have been widely used as models of BPD [7, 8]. At term, rodents are in the early saccular phase of lung development and display histological features of premature infants born at less than 30 weeks of gestation [11], albeit they have fully functional lungs. On the contrary, premature rabbits suffer from respiratory distress at birth, although they are also delivered in the early saccular phase. We, therefore, hypothesized that such functional differences at the starting point of both models (i.e., PND0 in mice and F28 in premature rabbits) would be reflected by the regulation of distinct molecular pathways. The comparison between term mice and rabbits showed that normal physiological birth activated common pathways in both species, despite term mice being in the saccular phase and premature rabbits in the alveolar phase. Interestingly, at the starting point of both BPD models, different gene sets were dysregulated in each specie. For instance, term mice showed an upregulation of pathways related to vascular development, whereas these genes were neither upregulated at F28 (Fig. 7B) nor in the first week of postnatal life following premature delivery (Fig. 6C). These findings suggest different molecular maturation degrees at the starting point of both BPD models, which may partly explain different responses to lung injury [62, 63] and pharmacological interventions [16, 64].

The present study has some limitations. In the first place, although we identified many pathways that are known to be involved in the development of BPD, we could not perform a head-to-head comparison with human data. Secondly, we conducted bulk transcriptomics on lung homogenates, which contain the transcripts of all lung cell types. Therefore, we could neither identify the changes nor the role of specific cell types at each developmental stage. Future studies applying single-cell transcriptomics [26] on the BPD rabbit model and on human samples would represent a significant advance in understanding the molecular pathways driving lung development in health and disease and may reveal novel therapeutic targets. In the present study, term rabbit pups were naturally delivered and mother-reared, whereas premature pups were delivered via C-section and fed milk formula, as would be expected in a clinical setting. We acknowledge that differences in delivery and postnatal care may have influenced our transcriptomic analysis. Nevertheless, the recent study by Salaets et al. [12] confirmed a significant impairment of lung development in premature rabbits compared to age-matched term rabbits, even when both term and preterm pups were delivered via C-section and fed with milk formula. Lastly, our analysis of the impact of premature birth was limited to one week of postnatal observation. Since the number of dysregulated genes increases along the first week of life in premature rabbits compared with age-matched term pups, studies are warranted to investigate the transcriptomic profiling and the pulmonary outcomes of premature rabbits at longer time points.

## Conclusion

We characterized the rabbit’s normal lung development using histological, transcriptomic and proteomic analyses, and investigated the impact of premature birth on the molecular regulation of this process. Histological findings corroborated that the rabbit’s lung development closely resembles the process in humans, showing developmental stage-specific morphological features and the intrauterine initiation of alveolarization. The time-resolved transcriptomic profile demonstrated the high translational power of the 28-day gestation premature rabbit as a model of BPD. At 28 days of gestation (F28), premature rabbits are delivered in the saccular phase, which concurs with the upregulation of genes and pathways involved in the pathophysiology of BPD, many of them essential to specialize the lungs for the extrauterine transition (e.g., angiogenesis and epithelium morphogenesis pathways). The analysis also revealed a significant impact of premature birth per se, without further perinatal insults (e.g., hyperoxia, LPS-induced inflammation), on the dysregulation of inflammatory and other pathways relevant for normal lung development (e.g., blood vessel morphogenesis and epithelial-mesenchymal transition). Altogether, these findings postulate the premature rabbit model, with or without additional insults, as a complementary alternative to rodent models for early stage mechanistic and pharmacological studies in the context of BPD.

## Supplementary Information

**Additional file 1:** Sequencing statistics and pathway analysis during normal rabbit lung development and after premature delivery. The first sheet (*sequencing stats*) contains the sequencing statistics. Sheets 2 (*Development Pathway analysis*) and 3 (*Pre-term Pathway analysis*) contain the pathway analysis of the rabbit’s normal lung development and the pathways analysis alterations due to premature delivery at 28 days of gestation, respectively.

## Data Availability

The datasets of the current study are available from the corresponding author on reasonable request.

## Abbreviations

BPD: Bronchopulmonary dysplasia
C-Section: Caesarean section
CAT: Catalase
COL1A1: Collagen Type I Alpha 1 Chain
COL1A2: Collagen Type I Alpha 2 Chain
CPM: Counts per million
CTNNB1: Catenin Beta 1
DDA: Data-dependent acquisition
DEG: Differentially expressed genes
E: Embryonic
ED: External diameter
F: Fetal
FASP: Filter-aided sample preparation
FC: Fold change
GEO: Gene expression omnibus
GPX1: Glutathione peroxidase
HCD: Higher energy collisional dissociation
HDAC1: Histone deacetylase 1
HDAC2: Histone deacetylase 2
ID: Internal diameter
IRS: Internal reference scaling
ME: Module eigengenes
MT%: Medial thickness of pre- and intra-acinar arteries
NF-κB: Nuclear factor κB
P: Postnatal
PCA: Principal component analysis
PDGFRB: Platelet derived growth factor beta
PECAM: Platelet and endothelial cell adhesion molecule 1
PND: Postnatal day
PRDX1: Peroxiredoxin 1
PRDX3: Peroxiredoxin 3
PRDX6: Peroxiredoxin 6
RAC: Radial alveolar count
RDS: Respiratory distress syndrome
ROS: Reactive oxygen species
SFTPA1: Surfactant protein A
SFTPB: Surfactant protein B
SOD1: Superoxide dismutase 1
T: Term
TD: Tissue density
TGF-β: Transforming growth factor- β
TGFBI: Transforming growth factor beta induced
TMM: Trimmed mean of M values
TMT: Tandem mass tags
TNF-α: Tumor necrosis factor-α
TXN: Thioredoxin
VEGF: Vascular endothelial growth factor
WGCNA: Weighted gene co-expression network analysis
WSI: Whole slide images
